# Overexpression of miRNA-21 promotes radiation-resistance of non-small cell lung cancer

**DOI:** 10.1186/1748-717X-8-146

**Published:** 2013-06-19

**Authors:** Wang Xiao-chun, Wang Wei, Zhang Zhu-Bo, Zhao Jing, Tan Xiao-Gang, Luo Jian-Chao

**Affiliations:** 1 Tianjin Key Laboratory of Molecular Nuclear Medicine, Institute of Radiation Medicine, Chinese Academy of Medical Science, Tianjin 300192, China; 2 Department of Radiation Oncology, Henan People’s Hospital, Henan 450003, China; 3 Department of Thoracic Surgery, Peking Union Medical College, Chinese Academy of Medical Sciences, Beijing 100021, China

**Keywords:** miRNA-21, NSCLC, Radiation-resistancet, Prognosis

## Abstract

**Background:**

MiRNA-21 was previously reported to be up-regulated in many kinds of cancer. In the present study, we want to investigate the potential role of miRNA-21 in non-small cell lung cancer.

**Materials and methods:**

Expression of miRNA-21 was detected in 60 non-small cell lung cancer (NSCLC) samples and adjacent histologically normal tissue using RT-qPCR, Correlation between miRNA-21 expression and clinicopathological features of NSCLC was analyzed using statistical software. The effect of miRNA-21 expression on the growth and apoptosis of A549 cells induced by irradiation was examined.

**Results:**

miRNA-21 expression increased in non-small cell lung cancer. Expression of miRNA-21 was positively associated with lymph node metastasis, clinical stage and poor prognosis. Multivariate Cox regression analysis showed that miRNA-21 was an independent prognostic factor for patients. Down-regulation of miRNA-21 inhibited proliferation and cell cycle progress of A549 cells and sensitized cells to radiation. Decreased miRNA-21 expression promoted the apoptosis of A549 cells induced by irradiation.

**Conclusions:**

miRNA-21 may be considered as a potential novel target for future development of specific therapeutic interventions in NSCLC.

## Introduction

Worldwide there are >1,600,000 new cases of lung cancer and >1,370,000 attributable deaths each year, ranking it as a leading cause of cancer mortality. In China lung cancer is the highest incidence of malignant tumor and the age-standardized incidence rate and mortality rate adjusted for the world standard population are both higher than the average levels worldwide [1].

MicroRNAs (miRNAs) are a class of small, evolutionary conserved RNA molecules that negatively regulate gene expression at the post-transcriptional level. The discovery of these small non-coding transcripts broadened our understanding of the mechanisms that regulate gene expression, added an entirely novel level of regulatory control. miRNAs consist of 18–25 nucleotides and are a class of endogenous ribo-regulators that modulate gene expression *via* the RNA interference (RNAi) pathway. Primary transcripts of miRNAs (pri-miRNA) are generated by RNA polymerase II [2], after which they are sequentially processed by RNase III class enzymes, Drosha and Dicer, to first produce ~70 nt-long intermediate hairpin structures (pre-miRNAs) and finally the mature miRNAs. MiRNAs act by base-pairing with their target mRNAs through perfect or nearly perfect complementarity at the 3′ untranslated regions (UTRs) of the target mRNAs leading to their translational repression and/or direct cleavage [3,4]. However, in some cases miRNAs can enhance mRNA translation. MiRNA-10a was found to bind the 5′UTR of ribosomal protein mRNAs and enhanced their translation, and some miRNAs were shown to switch from translation repression to promotion in a cell cycle-dependent manner [5,6]. Human miRNAs have been reported and a number of these have been shown to play normal physiologic roles in cell proliferation, apoptosis, and differentiation [7]. In addition, studies have showed that miRNAs contributed to oncogenesis by promoting the expression of oncogenes or by inhibiting tumor suppressor genes. As such, some miRNAs may be markers for cancer diagnosis and prognosis [8].

MiRNA-21was one of the first miRNAs to be identified as transcribed by RNA polymerase II, which subsequently has been identified as a major driver of miRNA transcription. The gene coding for pri-miRNA-21 (primary transcript containing miRNA-21) is located within the intronic region of the TMEM49 gene. Despite pri-miRNA-21 and TMEM49 are overlapping genes in the same direction of transcription, pri-miRNA-21 is independently transcribed by its own promoter regions and terminated with its own poly (A) tail. After transcription, pri-miRNA-21 is finally processed into mature miRNA-21[9]. MiRNA-21 has been shown to be overexpressed in multiple malignancies including pancreatic cancer [10,11], esophageal cancer [12], lung cancer [13], and colon cancer [14]. This miRNA has been linked to tumor aggression and carcinogenesis, in part, by preventing apoptosis and, thus, functioning as an oncogene [15,16]. In the present study, we examined the expression of miRNA-21 in non-small cell lung cancer (NSCLC) and explored its effects on radio-sensitivity of NSCLC. The results indicated that miRNA-21 was overexpressed, and was associated with lymph node metastasis and poor prognosis of NSCLC. Moreover, its overexpression promoted the radio-resistance of NSCLC cells.

## Materials and methods

### Patients and sample

Sixty fresh tissue samples, containing NSCLC and adjacent histologically normal tissue, were procured from surgical resection specimens collected by the Department of Tumor Medicine, Henan People’s Hospital from 2001 to 2007. Primary tumor regions and corresponding histologically normal tissues from the same patients were separated by experienced pathologists, and immediately stored in liquid nitrogen (–193°C) until use. All patients were received no treatment before surgery and signed informed consent forms for sample collection. Use of patient samples comprising tumor and adjacent histologically normal tissues was approved by our institutional ethics committee of Radiation Medicine Institute. For all the samples, clinic-pathological information (smoking, age, gender, pathological subtype, TNM classification, tumor stage, lymph node stage, differentiation status and the duration of survival after surgery) was available.

### Cells culture and ionizing radiation

Lung cancer A549 cells were cultured in RPMI 1640 (Invitrogen) supplemented with 10% fetal bovine serum (FBS) at 37˚C under 5% CO2 in a humidified incubator. Cells were exposed different dose of irradiation in a JL Shepherd Model 143 ^137^Cesium γ-irradiated at a rate of 2.4 Gy/min.

### RNA extraction

Total RNA was extracted from NSCLC tissue and its corresponding normal tissue using the Absolutely RNA™ RT-PCR Miniprep kit (Stratagene), according to the manufacturer’s instructions. Total RNA concentration was adjusted to 2 ng/μl using a spectrophotometer.

### Real-time RT-PCR quantification of miRNA-21

Taq Man miRNA assays (ABI PRISM) used the stem-loop method to detect expression levels of mature miRNA-21[17]. For reverse transcription (RT) reactions, 10 ng total RNA was used in each reaction (5 μl) and mixed with RT primer (3 μl). RT reactions were carried out at 16°C for 30 min, 42°C for 30 min and 85°C for 5 min, then maintained at 4°C. Following RT reactions, 1.5 μl cDNA was used for a polymerase chain reaction (PCR) along with Taq Man primers (2 μl). PCR was conducted at 95°C for 10 min followed by 40 cycles at 95°C for 15 sec and at 60°C for 60 sec in the ABI 7500 real-time PCR system. Real-time PCR results were analyzed and expressed as relative miRNA expression of the threshold cycle (CT) values. RT and PCR primers for miRNA-21 were purchased from ABI PRISM. U6B was used for normalization. Relative expression level between different treatments were then calculated using the following equation:   = 2^- (ΔCtsample-ΔCt control)^[17]. A two-fold change in either direction was considered to be significant.

### miRNA transfection and northern blots

The miRNA-21 RNAi vector was constructed by cloning of annealed oligonucleotides that contained the pre-miRNA-21 sequence into the pSuppressorNeo expression vector. A scrambled sequence without significant homology to any rat, mouse or human gene was used as a negative control (scrambled group). Transfection was performed using Lipofectamine™ 2000 (Invitrogen, Carlsbad, CA) according to the manufacturer’s instructions. Briefly, one day before transfection, cells was plating in 500 μl of growth medium without antibiotics so that cells will be 90-95% confluent at the time of transfection. 50 nM DNA was diluted in 50 μl medium without serum. Mixing Lipofectamine™ 2000 gently before use, then diluting 5 μl in 50 μl medium. Incubating for 5 min at room temperature. Then the diluted DNA was combined with the diluted Lipofectamine 2000. Mixing gently and incubating for 20 min at room temperature. Adding the complexes to each well containg cells and medium. The plate was mixed gently by rocking back and forth. Incubating the cells at 37°C in CO_2_ incubator for 48 h. Then northern blots assay was used to confirm the knock-down effects of miRNA-21 expression.

For miRNA northern blots, 15 μg of total RNA were separated on 15% denaturing polyacrylamide gels, electrotransferred onto GeneScreen Plus membranes (PerkinElmer), and hybridized using UltraHyb-Oligo buffer (Ambion). Oligonucleotides complementary to mature miRNA-21(5′-aucgaauagucugacuacaacu-3′) were end-labeled with T4 Kinase (Invitrogen) and used as probes. Hybridization was performed at 42°C overnight and membranes washed twice in 0.1×SSPE and 0.1% SDS at 42°C for 15 min each. Membranes were then exposed to a storage phosphor screen (GE Healthcare Bio-Sciences) for 8 h and imaged using a Typhoon 9410 Variable Mode Imager (GE Healthcare Bio-Sciences). Saved images were cropped using Photoshop 6.0 (Adobe Systems).

### Cell proliferation assay

Four hundred cells were aliquoted into each well of a 6-well plate in triplicate and exposed to γ-ray with different dose of irradiation. After 10 days of incubation, the colonies were stained with the Giemsa stain and a minimum of 50 viable cells were counted. Quantity One software (version 4.6.2) was used to analyze the results.

The effects of miRNA-21 expression on A549 cell proliferation were assessed using the Cell Counting Kit-8 (CCK-8, Dojindo, Japan). Briefly, the cells were plated in 96-well plates. Cells was irradiated with 6 Gy γ-ray in 48 hours after transfection. Then CCK-8 (10 μl) was added to each well at various time points and incubated at 37°C for 1.5 h. Cells were harvest at 1, 2, 3, 4, 5 and 6 day after irradiation, respectively. The absorbance at 450 nm was measured using a microplate spectrophotometer.

### Cell cycle assay

Cell cycle profiles were examined using flow cytometry. Briefly, cell monolayers were washed with phosphate-buffered saline (PBS), trypsinized and resuspended in ice-cold PBS. Cells were then gently pelleted by centrifugation (300×*g* for 5 min at 4°C), the supernatant was removed and the cells were fixed and permeabilized by the dropwise addition of 70% ethanol at -20°C while vortexing. Fixed cells were washed with PBS and incubated in the dark for 30 min with a propidium iodide (PI) staining solution containing 50 μg/ml propidium iodide (PI) and 100 μg/ml RNase A in PBS. The propidium iodide fluorescence per cell was measured with a Flow Cytometer (BD, Franklin Lakes, NJ) equipped with a 488 nm argon laser. The width (FL2W) and area (FL2A) of the PI fluorescence per cell were recorded for at least 10,000 cells per sample. Histograms of the PI intensities were plotted. The percentage of cells in each phase of the cell cycle was analyzed using ModFit software.

### Cell apoptosis assay

Cells were harvested 8 hours post irradiation with a dose of 0, 4 and 8 Gy for apoptosis detection using the annexin V-FITC apoptosis detection kit (Sigma) and subsequently analyzed by flow cytometry.

### Western blot analysis

Whole cell extracts were prepared from cultured cells by homogenizing cells in a lysis buffer (10 mM Tris–HCl (pH 7.5), 150 mM NaCl, 1% NP40) containing a cocktail of protease inhibitors. After centrifugation at 15,000 RCF for 30 min at 4°C, supernatants were recovered and used for immunoblot analysis. The proteins were separated by SDS-PAGE and then transferred to polyvinylidene difluoride (PVDF) membranes (Millipore). Blots were blocked and then probed with antibodies against Pro-caspase3 (1:500 dilution, Santa Cruz), PARP (1:500, Santa Cruz) and β-actin(1:1000, Santa Cruz). After washing, the blots were incubated with horseradish peroxidase-conjugated secondary antibodies and visualized by super ECL detection reagent (Applygen, Beijing, China).

### Statistical analysis

All statistical analysis was performed using SPSS16.0 software. Results were statistically evaluated using Chi-square test. Patient survival curves were estimated by the Kaplan-Meier method. The median miRNA intensity value of the initial training cohort was used as the cut point in Kaplan-Meier survival analysis, and patients were categorized into groups with high (above median) or low (below median) expression. The joint effect of covariables was examined using the Cox Proportional Hazard Regression Model. *p* < 0.05 was considered to be statistically significant.

## Results

### MiRNA-21 expression level was higher in NSCLC tissue than in corresponding non-cancerous tissue

**Figure 1:**
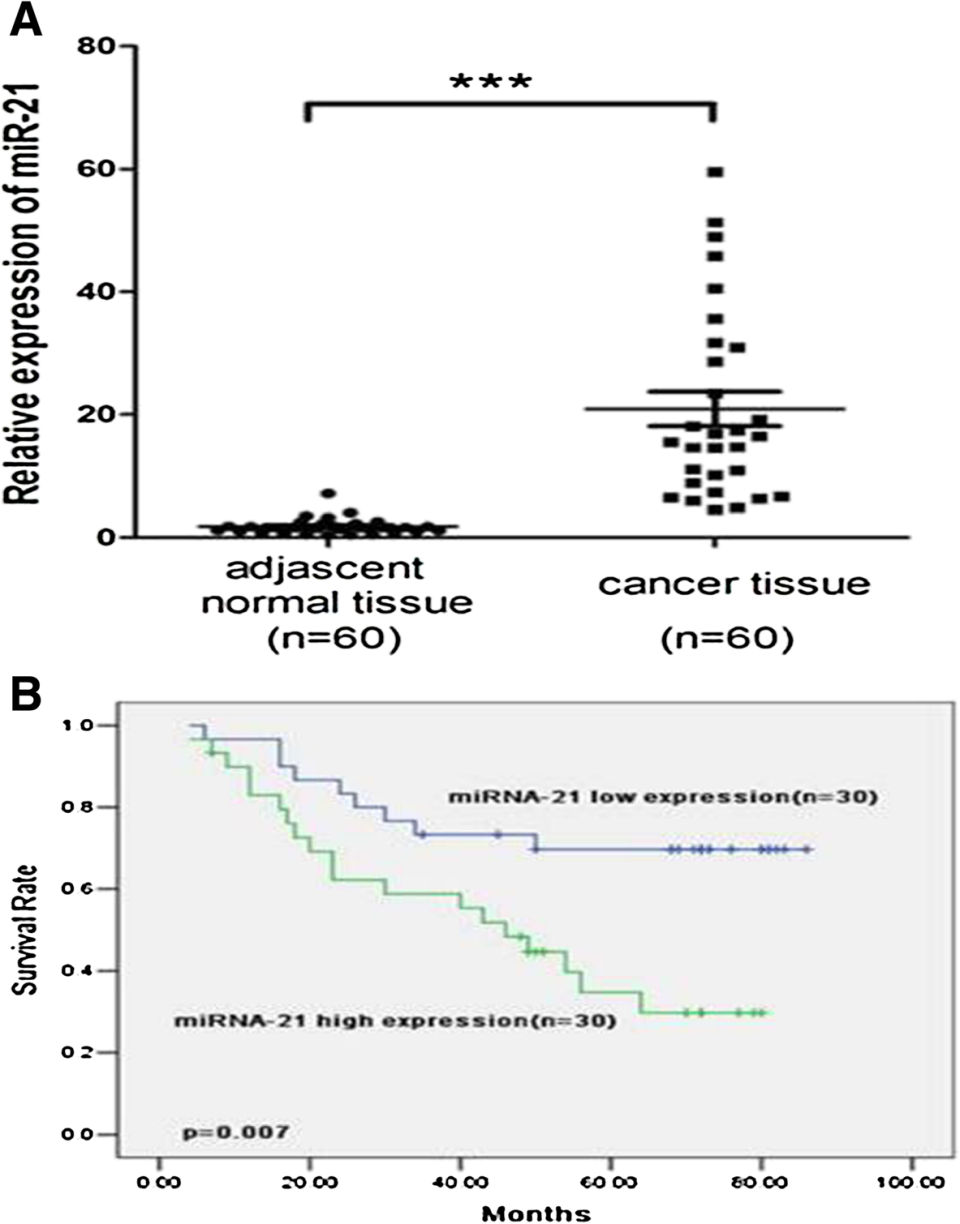
**Expression of miRNA-21in NSCLC and its correlation with prognosis of patients. A**, miRNA-21 expression in NSCLC cancer patients detected by real-time RT-PCR. MiRNA-21 expression level was higher in NSCLC tissue than in corresponding non-cancerous tissue. (*p*<0.0001). U6 snRNA was used as an internal control. Horizontal line, mean value of each group. *** *p*<0.0001. **B**, Kaplan-Meier survival curve of NSCLC patients sub-grouped as miRNA-21 low or high expression. The prognosis of patients with high miRNA-21 was significantly shorter than that of patients with miRNA-21 low patients (*p*=0.007).

Expression of miRNA-21 was detected in 60 NSCLC samples and adjacent histologically normal tissues using RT-qPCR, and its expression was normalized to that of the control U6B small nuclear RNA gene (RNU6B). Results showed miRNA-21 expression levels were significantly higher in NSCLC tissues than that in corresponding non-cancerous tissues (Figure 1A). Statistical analysis showed that overexpression of miRNA-21 was associated with lymph node metastasis and clinical stage of NSCLC (Table 1). To determine the association between miRNA-21 expression and prognosis, Kaplan–Meier curves for overall survival was plotted. Significant difference was observed in NSCLC patient survival according to miRNA-21 expression in tumor tissues. The survival rate of patients with low miRNA-21 expression was higher than that of patients with high miRNA-21 expression (*p*=0.007) (Figure 1B). Multivariate Cox regression analysis showed that miRNA-21 expression (*p*=0.032), regional lymph node metastasis (*p*=0.015) and clinical stage (*p*=0.004) were independent prognostic factors for NSCLC patients (Table 2).

**Table 1:** Relationship between miRNA-21 expression and tumor clinicopathologic features

**Clinicopathologic features**	**Number of cases**	**miRNA-21 expression**		
		**Low (n=19)**	**High (n=41)**	***P***
**Age, years**				
≥60	38	10	28	0.242
<60	22	9	13	
**Sex**				
Male	34	12	22	0.490
Female	26	7	19	
**Smoking**				
Smoking	33	11	22	0.759
No smoking	27	8	19	
**Lymph node metastasis**				
N0	29	15	14	0.005
N1	31	4	27	
**Stage**				
I + II	29	14	15	0.007
III +IV	31	5	26	
**Grade**				
G1+ G2	32	10	22	0.941
G3	28	9	19	

**Table 2:** Postoperative survival of patients in relation to clinicopathological characteristics and microRNA expression analyzed by the Cox proportional hazard regression model in 60 cases

**Variables**		**Univariate analysis**	**Multivariate analysis**
		**Hazard ratio (95% CI) p**	**Hazard ratio (95% CI) p**
Smoking	+/-	1.014(0.385-2.668) 0.978	1.351(0.477-3.831) 0.571
Age	≥60	1.111(0.528-2.338) 0.456	1.053(0.478-2.324) 0.897
Sex	M/F	1.265(0.481-3.331) 0.634	1.710(0.595-4.915) 0.319
LN metastasis	+/-	2.389(1.099-5.196) 0.025	2.045(1.072-3.513) 0.015
TNM	I+II/III	1.794(1.131-2.845) 0.003	4.021(1.764-6.462) 0.004
Differentitation	Well+mod/poor	1.283(0.656-2.508) 0.654	1.687(0.734-3.879) 0.218
miRNA-21	Low/High	2.827(1.274-6.275) 0.011	2.103(0.695-3.078) 0.032

*LN* Lymph node, *TNM* Stage of Tumor, Regional Lymph node and Metastasis; *CI* Confidence Interval.

### Knock-down of miRNA-21 promoted the radio-sensitivity of A549 cells

**Figure 2:**
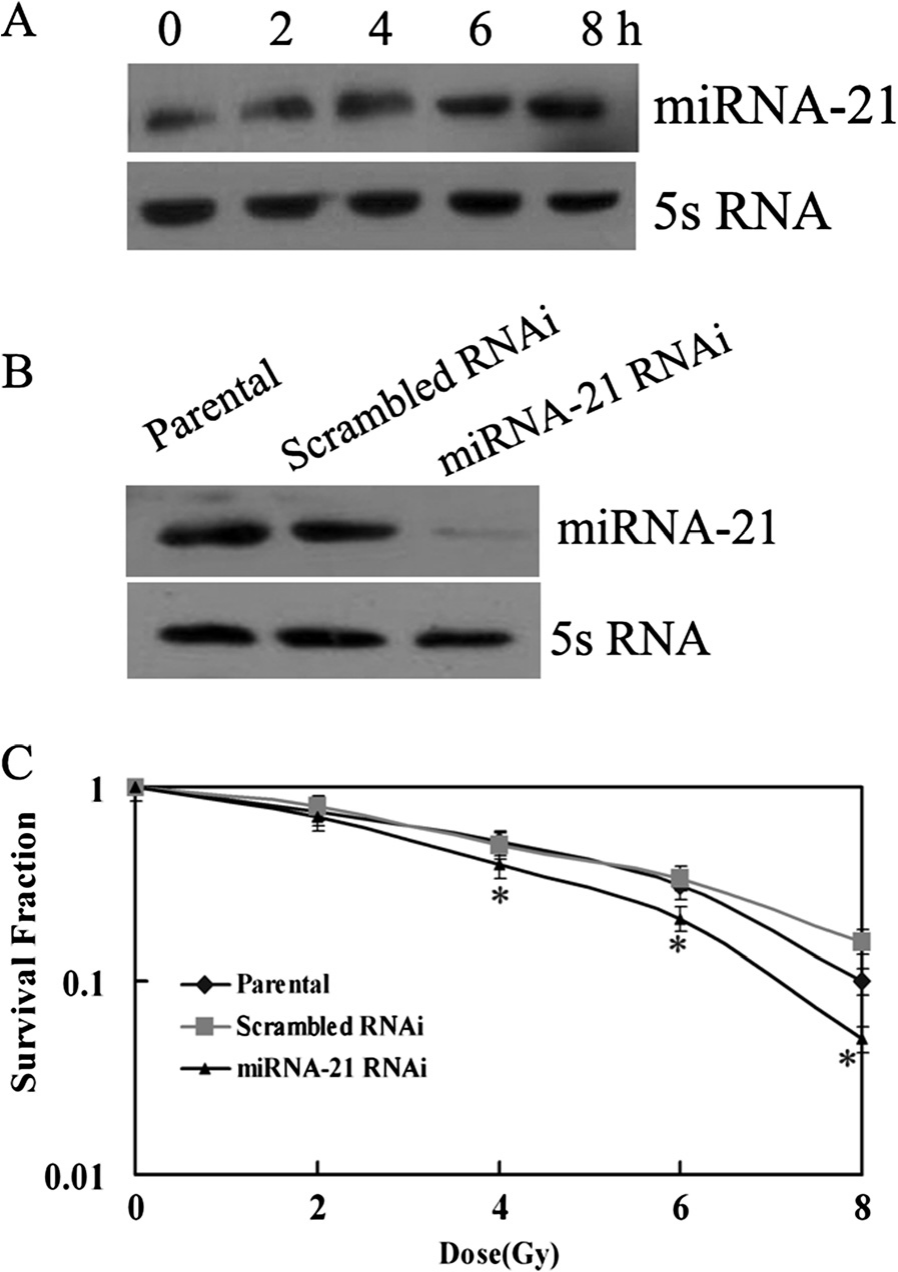
**Effects of miRNA-21 expression on radio-sensitivity of NSCLC cell. A**, Elevated miRNA-21 was observed after γ- ray radiation with a dose of 6 Gy, and its level was highest at 8 hours. **B**, A549 cells were transfected with miRNA-21 RNAi and scrambled RNAi vector. Expression of miRNA-21 was detected in 48 hours after transfection miRNA-21 level decreased in RNAi cells. **C**, Cells were exposed to various doses of IR (0 Gy, 2 Gy, 4 Gy, 6 Gy and 8 Gy) at a rate of 2.4 Gy/min 48 hours after transfection, then clonogenic assay was performed. Knock-down of miRNA-21 promoted the radio-sensitivity of A549 cells,*p<0.05.

MiRNA-21 expression was examined the in A549 cells at 0, 2, 4, 6 and 8 hours after radiation with a dose of 6Gy. MiRNA-21 expression was increased after radiation. Its level increased after 4 hours, showing that miRNA-21 may have effects on the radiation response of A549 cells (Figure 2A).

### Knock down miRNA-21 inhibited growth of A549 cells induced by gamma radiation

To determine whether miRNA-21 actually enhances the sensitivity of A549 cell lines to radiation-induced death, A549 cells were transfected with miRNA-21 RNAi and scrambled RNAi vector. 48 hours after transfection, the cells were exposed to various doses of IR (0, 2, 4, 6 and 8 Gy) at a rate of 2.4Gy/min. Then clonogenic assay was performed. Results showed that the survival fraction of miRNA-21 knock-down cells was significantly lower than that of control groups (Figure 2C), showing a correlation between miRNA-21 expression and radio-sensitivity of A549 cells to gamma radiation.

**Figure 3:**
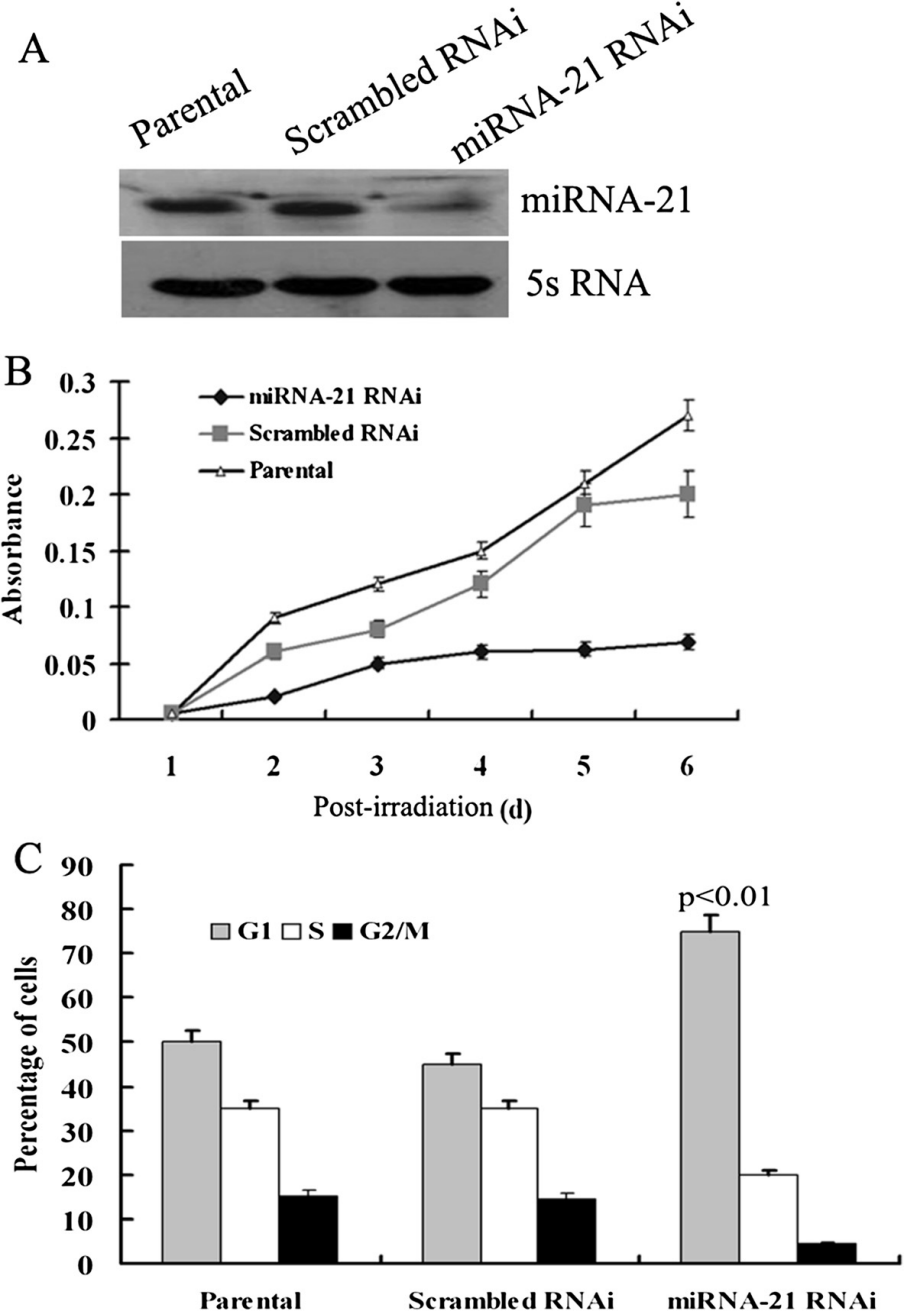
**Down-regulation of miRNA-21 inhibited A549 cell proliferation and cycle progress after IR. A**, A549 cells were transfected with miRNA-21 RNAi and scrambled RNAi vector. Expression of miRNA-21 was detected in 48 hours after transfection. miRNA-21 level decreased in RNAi cells. **B**, Statistical plots of 3 independent cell growth assay.A549 cells were irradiated with 6 Gy γ-ray in 48 h after transfection. Then cells were harvest at 1, 2, 3, 4, 5 and 6 day after irradiation, respectively and cell growth was monitored. The proliferation ability of miRNA-21 knock down cells was lower than that of control groups. **C**, Cells were exposed to 6 Gy γ-ray in 48 hours after transfection. Then these cells were harvested and cell cycle was examined by flow cytometry. Percentage of cells in different cell cycle phases was plotted and results were represented as mean ± s.d. Comparing with the control groups, the percentage of cells at G1 phase was significantly increased, whereas the population of cells at S phase significantly decreased in miRNA-21 knock down group (*p*<0.01).

We also examined the effects of miRNA-21 expression on cellular proliferation after exposed to 6 Gy IR. The results showed that knock down of miRNA-21 inhibited the growth of A549 cells (Figure 3A and B).

### Knock down miRNA-21 affected the cell cycle of A549 cells induced by gamma radiation

Cell cycle analysis was executed to determine whether the effect of miRNA-21 on cell proliferation was due to cell cycle alterations. A549 cells were transfected with miRNA-21 RNAi and scrambled RNAi vector. 48 hours after transfection, cells were exposed to 6 Gy γ-ray. Then these cells were harvested and cell cycle was examined by flow cytometry. The result showed that as comparing to the control group, the percentage of cells at G1 phase was significantly increased, whereas the population of cells at S phase significantly decreased in miRNA-21 knock down group(*p*<0.01) (Figure 3C). These results indicated that decreased expression of miRNA-21 induced G1 cell cycle arrest in A549 cells.

### Knock down miRNA-21 promoted the apoptosis of A549 cells induced by gamma radiation

**Figure 4:**
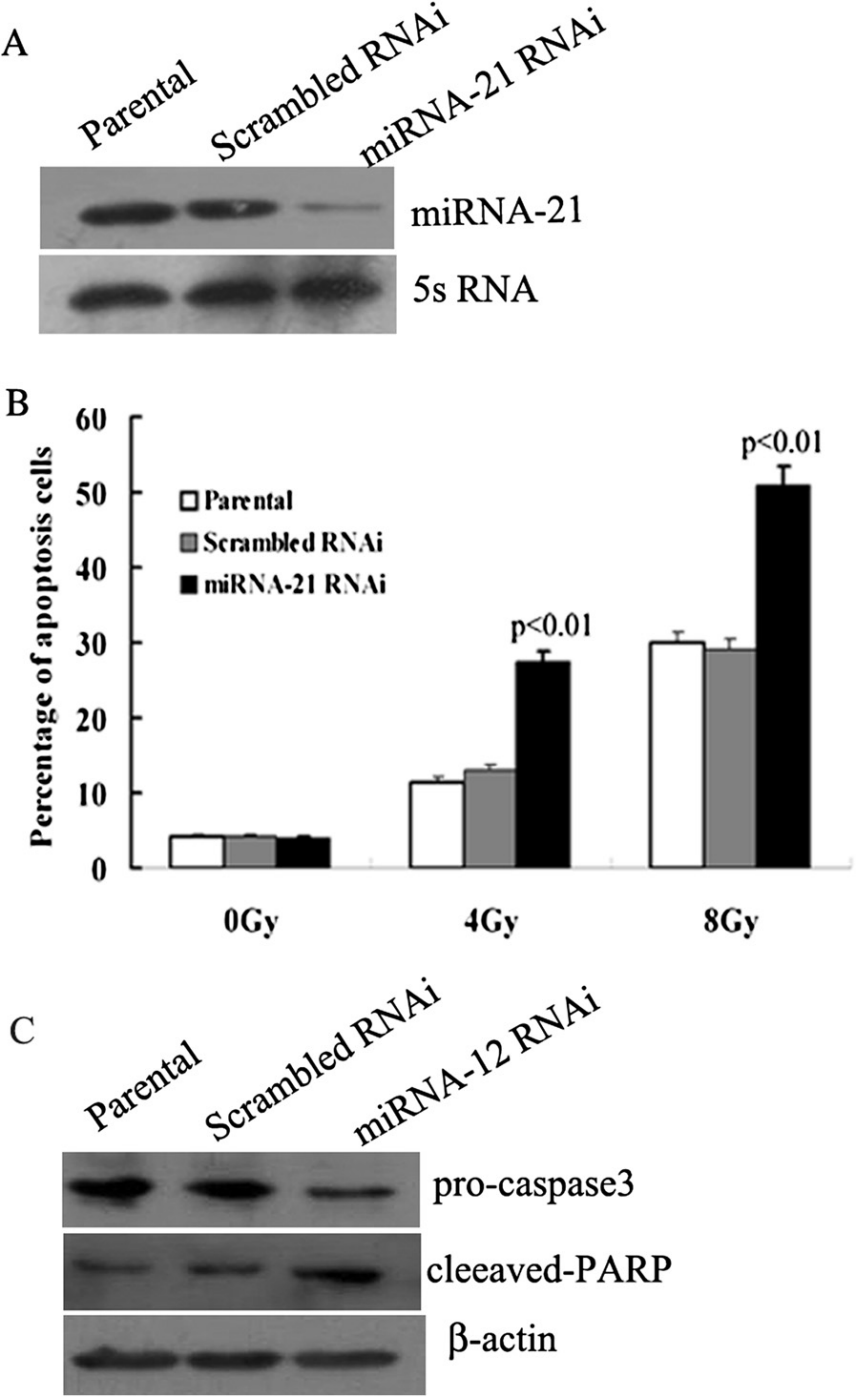
**Effects of miRNA-21 knock down on the apoptosis of A549 cells induced by gamma radiation. A**, Expression of miRNA-21 decreased in miRNA-21 RNAi cells. **B**, Detection of apoptosis induced by various dose of irradiation through annexin V-FITC/PI staining and results were ploted as mean ± s.d. from triplicate experiments. *p*<0.01 *vs* parental and scrambled RNAi groups. Percentage of apoptosis in miRNA-21 knock-down group were significantly higher than that of control groups in both 4 and 8 Gy dose. **C**, Down-regulation of miRNA-21 led to increased activation of Caspase 3, as evident from increased levels of Cleaved-PARP.

We next explored its role in the apoptosis of NSCLC cells induced by irradiation. A549 cells were transfected with miRNA-21 RNAi and scrambled RNAi vector, 48 hours later, these cells were exposed to various doses of IR (0, 4 and 8 Gy) at a rate of 2.4Gy/min. As shown in Figure 4A, miRNA-21 level decreased after transfected with miRNA-21 RNAi vector. The percentage of apoptosis cells in miRNA-21 knock-down group was significantly higher than that of parental and control group at the dose 4 and 8 Gy (*p*<0.05). At the dose of 0 Gy, no significantly different was found among these three groups in the percentage of apoptosis cells (Figure 4B). We then examined the effects of miRNA-21 knockdown on caspase 3 activity (a central mediator of apoptosis) after 4 Gy IR. Decreased miRNA-21 expression markedly increased the activity of caspase 3 compared to parental or scrambled RNAi controls (Figure 4C).

## Discussion

Studies have shown that miRNAs expression fingerprints correlated with clinical and biological characteristics of tumours, including tissue type, differentiation, aggression, response to therapy and prognosis [18]. A large amount of diagnostic information was encoded in a relatively small number of miRNAs. Amplification or overexpression of an oncogenic miRNA could eliminate the expression of a miRNA-target tumor suppressor gene, and result in cancer progression [19]. MiRNA-21 has been classed as an oncogenic miRNA and its overexpression could lead to tumor development and progression. Up-regulation of miRNA-21 has been found in numerous human cancers. Markou et al. found overexpression of mature miRNA-21 was an independent negative prognostic factor for overall survival in NSCLC patients [20]. Wang et al. found that the level of miRNA-21 expression was higher in NSCLC serum samples than in control serum samples. High serum miRNA-21 was significantly correlated with tumor-node metastases stage and lymph node metastasis of NSCLC patients. The 3-year actuarial overall survival rate in NSCLC patients with high serum miRNA-21 expression was significantly shorter than those with low serum miRNA-21 expression. Moreover, serum miRNA-21 expression was an independent prognostic factor for NSCLC patients [21]. Shen et al. also reported that altered expression of the miRNAs (miRNA-21, -126,-201 and 486-5p) in plasma would provide potential blood-based biomarkers for NSCLC [22]. In the present study, we found that miRNA-21 expression levels were significantly higher in NSCLC tissues and its expression was associated with lymph node metastasis and clinical stage. In addition, miRNA-21 also was an independent prognostic factor for NSCLC patients. Both our and others results showed that miRNA-21 might be a useful diagnostic and prognostic marker for NSCLC patients. A non-transcriptional mechanism for miRNA-21 upregulation implying gene amplification, rather than promoter hyper-activation, has been proposed [23]. However, most of the available data suggest that miRNA-21 expression is maintained by transcriptional and post-transcriptional regulation [24].

Although many studies have explored the role of miRNA-21 in cancer, there are few reports about the relationship between miRNA-21 expression and radio-sensitivity of cancer. Want *el al* detected the expression profile of miRNA in postoperative radiotherapy sensitivity and resistant patients of NSCLC. They found miRNA-21 was greatly down-regulated in radiotherapy sensitive patients [25]. Zhu et al. reported that miRNA-21 was the only one that increased 6 folds in high-LET (low linear energy transfer) radiation promoted mouse liver tumors when compared with that in the non-irradiated liver tissues. They also showed that miRNA-21 was up-regulated in human or mouse hepatocytes after exposure to radiation, as well as in liver tissues derived from whole body irradiated mice [26]. These results suggested that miRNA-21 expression may have effects on the radio-sensitivity of tumors. Given that radiation therapy is one of the most important methods for NSCLC treatment, we examined the effects of miRNA-21 expression on radio-sensitivity of A549 cells in the present study. The clonogenic formation assay showed that down-regulation of miRNA-21 promoted the radio-sensitivity of A549 cells. Moreover, decreased miRNA-21 level inhibited proliferation and cell cycle progress of A549 cells after IR. We did not further explore the potential mechanism. But it has been reported that miRNA-21 had the ability to suppress the expression of the tumor suppressor PTEN [27,28]. miRNA-21 also directly represses the expression of the tumor suppressor gene tropomyosin 1 (TPM1) [29]. These mechanisms may also be function in the condition of IR.

We also found that decreased miRNA-21 expression promoted the apoptosis of A549 cells induced by irradiation. At the molecular level, caspase-3 was activated in miRNA-21 knock down cells. It has been reported that down regulation of miRNA-21 in clultured glioblastoma cells triggered activation of caspases and led to increased apoptotic cell death [15]. These data suggested that aberrantly expressed miRNA-21 may contribute to the malignant phenotype and radio-resistance of tumors by blocking expression of critical apoptosis-related genes.

## Conclusion

In conclusion, our results revealed that miRNA-21 was overexpressed in NSCLC tumors. Increased expression of miRNA-21 was strongly associated with lymph node metastasis and poor prognosis. In addition, Down-regulation of miRNA-21 inhibited proliferation and cell cycle progress of A549 cells and sensitized cells to radiation. Our findings provide a new role of miRNA-21 in NSCLC, and it may be considered as a potential novel target for future development of specific therapeutic interventions in NSCLC.

## Competing interests

The authors declare that they have no competing interests.

## Authors’ contributions

WW and ZZ-B carried out the molecular biology studies. ZJ carried out the cell biology studies. TX-G and LJ-C collected the samples. WX-C designed this study. All authors read and approved the final manuscript.
